# Decreased performance in IDUA knockout mouse mimic limitations of joint function and locomotion in patients with Hurler syndrome

**DOI:** 10.1186/s13023-015-0337-3

**Published:** 2015-09-25

**Authors:** Chihwa Kim, Min Jung Kwak, Sung Yoon Cho, Ah-ra Ko, Jinguen Rheey, Jeong-Yi Kwon, Yokyung Chung, Dong-Kyu Jin

**Affiliations:** Clinical Research Center, Samsung Biomedical Research Institute, Seoul, Republic of Korea; Department of Pediatrics, Pusan National University Hospital, Pusan National University School of Medicine, Busan, Republic of Korea; Department of Pediatrics, Samsung Medical Center, Sungkyunkwan University School of Medicine, 81 Irwon-ro, Gangnam-gu, Seoul, 135-710 Republic of Korea; Samsung Biomedical Research Institute, Samsung Advanced Institute of Technology, Seoul, Republic of Korea; Department of Physical and Rehabilitation Medicine, Sungkyunkwan University School of Medicine, Seoul, Republic of Korea; Department of Molecular Science and Technology, Ajou University, Suwon, Republic of Korea; Present Address: MOGAM Biotechnology Institute, Yongin, Republic of Korea

**Keywords:** IDUA, IDUA KO mice, BMD, Rotarod test, Open-field test

## Abstract

**Background:**

Mucopolysaccharidosis type I (MPS I) is caused by the deficiency of alpha-L-iduronidase (IDUA), which is involved in the degradation of glycosaminoglycans (GAGs), such as heparan sulfate and dermatan sulfate in the lysosome. It has been reported that joint symptoms are almost universal in MPS I patients, and even in the case of attenuated disease, they are the first symptom that brings a child to medical attention. However, functional tests and biological markers have not been published for the evaluation of the limitations in joint and locomotion in animal model-mimicking MPS.

**Methods:**

We generated IDUA knockout (KO) mice to observe whether they present impairment of joint function. KO mice were characterized phenotypically and tested dual-energy X-ray absorptiometry analysis (DEXA), open-field, rotarod, and grip strength.

**Results:**

The IDUA KO mice, generated by disruption between exon 6 and exon 9, exhibited clinical and laboratory findings, such as high urinary GAGs excretion, GAGs accumulation in various tissues, and significantly increased bone mineral density (BMD) in both female and male mice in the DEXA of the femur and whole bone. Remarkably, we observed a decrease in grasp function, decreased performance in the rotarod test, and hypo-activity in the open-field test, which mimic the limitations of joint mobility and decreased motor performance in the 6-min walk test in patients with MPS I.

**Conclusions:**

We generated a new IDUA KO mouse, tested open field, rotarod and grip strength and demonstrated decrease in grip strength, decreased performance and hypo-activity, which may be useful for investigating therapeutic approaches, and studying the pathogenesis of joint and locomotion symptoms in MPS I.

**Electronic supplementary material:**

The online version of this article (doi:10.1186/s13023-015-0337-3) contains supplementary material, which is available to authorized users.

## Background

Mucopolysaccharidosis type I (MPS I), known as Hurler syndrome [OMIM, #607014], is caused by a deficiency of IDUA lysosomal enzyme, and is inherited as autosomal recessive disease. This enzyme is involved in glycosaminoglycans (GAGs)’ metabolic pathway, and its deficiency causes GAGs accumulation in the lysosomes. MPS I has three subtypes depending on phenotypic involvement: MPS I-H (Hurler), the severe type; MPS I-S (Scheie), the mild type; and MPS I-HS (Hurler–Scheie) are intermediated in phenotypic expression [1]. MPS I-H is characterized by impaired cognitive development, progressive coarsening of the facial features, hepatosplenomegaly, respiratory failure, cardiac valve dysfunction, recurrent otitis media, corneal clouding, musculoskeletal manifestations, such as joint stiffness and contractures, and dysostosis multiplex. The symptoms arise after birth and progress rapidly [2]. MPS I-S, in which the symptoms occur later and progress slowly, is the most attenuated form of MPS I. While some individuals with this form have no neurologic involvement and psychomotor development may be normal in early childhood, learning disabilities can be present. Hearing loss and cardiac valvular disease are common. Death, typically caused by cardiorespiratory failure, usually occurs within the first ten years of life [2, 3]. MPS I-HS manifests in infancy with intermediate severity. The somatic symptoms reduce life expectancy to the second or third decade of life. Generally, there is no cognitive impairment, although some patients may exhibit mild learning difficulties [4].

Among its symptoms, musculoskeletal manifestations are an important clue for the diagnosis of MPS I. As the disease evolves, carpal tunnel syndrome, stiffening of the interphalangeal joint, hook finger, and gripped hand are common and impair hand function [5]. In particular, MPS has been known to be the most common cause of carpal tunnel syndrome in children [6]. Carpal tunnel syndrome is caused by the thickening of the flexor retinaculum and the tissues around the tendon sheaths. Additionally, surgical release of the tendons can improve hand function [7].

Some MPS I animal models have been developed or naturally occurred previously in felines [8, 9], canines [10–12], and mice [13–16]. The canine model was used in the efficacy test of enzyme replacement therapy (ERT) [10, 17, 18]. Two MPS I-H mouse models have been generated by gene disruption in exon 6 [13, 14], and they were shown to have similar phenotypes with those of MPS I-H patients. Another model was generated by knock-in that carries a nonsense mutation, IDUA-W392X, being introduced into exon 9, and they found correlations with other MPS I-H animal model characteristics, including decreased enzyme activity, the accumulation of GAGs in the urine, and lysosomal storage in many kinds of cells [15].

In addition to these animal models, in the present study we generated another MPS I-H mouse model with large deletion from exons 6 to 9 in the IDUA gene. We found that our IDUA knockout (KO) mice exhibited findings that are the same as those reported before, such as increased urinary and tissue GAGs and thick bone [13–16]. Moreover, we present detailed behavior functions of the mice, especially in relation to joint and locomotion function, which can be used in evaluating the efficacy of the therapeutic modalities in MPS I. Thus, we expect that the IDUA KO mouse model presented here will be a very useful tool for evaluating the efficacy of therapeutic approaches.

## Methods

### Ethics statement

This study was reviewed and approved by the Institutional Animal Care and Use Committee (IACUC) of the Samsung Biomedical Research Institute (SBRI). SBRI is an accredited facility by the Association for Assessment and Accreditation of Laboratory Animal Care International (AAALAC International) and abides by the Institute of Laboratory Animal Resources (ILAR) guidelines.

### Generation of IDUA KO mice

**Fig. 1:**
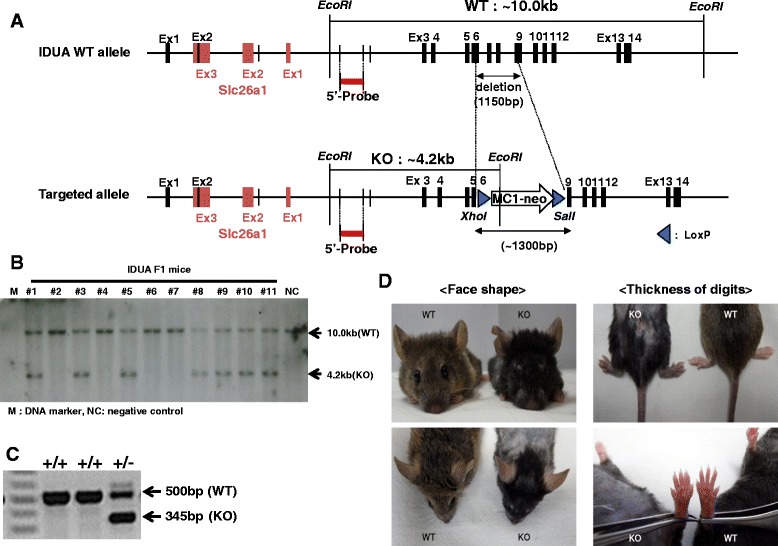
Generation of IDUA knockout mouse and gross photos of external morphology. **a** The wild-type allele (upper) of the IDUA gene consists of 14 exons, while the targeted allele (lower) includes exon 1 to a part of exon 6 and a part of exon 9 to 14. The IDUA KO construct (lower) was integrated into the mouse IDUA gene locus by homologous recombination. **b** Genomic DNA obtained from 11 F1 offspring mice were digested *EcoRI.* Southern blotting analysis was performed with 5’-probe (896 bp, hybridized to the left arm region of both alleles). Wild-type mice showed 10 Kbp, and the heterozygous type showed 10 and 4.2 Kbp. **c** PCR analysis of the IDUA gene was performed in tail genomic DNA to confirm the expected deletion of the IDUA gene. **d** The IDUA KO mouse showed a flattened facial profile, more blunt nose, less prominent eyes, rough fur, and thickening of the digits

The IDUA KO mouse model of Hurler syndrome was generated by replacing a part of the IDUA gene located on chromosome 5 (NC_0000701.5) in mice with the neomycin resistance gene. OSDupDel.Neo vector was used as expression vector (Open Biosystems, Alabama, USA), and the KO construct included intron 1 to a part of exon 6 of the IDUA gene (2534 bp) for the left arm, a region from a part of exon 9 to the outside of exon 14 for the right arm (7112 bp), and the neomycin resistance gene (1300 bp). pOSDupDel.Neo-IDUA construct was transfected into embryonic stem cells, and the positive cell clones were selected by Southern blotting and microinjected into C57BL/6 blastocysts. Targeted disruption of the mouse IDUA locus was performed by homologous recombination with the neomycin resistance gene. IDUA embryonic stem (ES) cell clones and F1 offspring were analyzed after *EcoRI* digestion of genomic DNA by Southern blotting with a 5’-probe consisting of 896 bp (Fig. 1a). The genomic DNA band size was 10 Kbp for the wild type and 4.2 Kbp for IDUA KO mice bred with a wild-type C57BL/6 strain. All offspring were genotyped using the PCR of tail genomic DNA. To perform the PCR screening of IDUA KO mice, four kinds of primers were used: TTCCAGACCCTGTTGGGTGGGC (forward) and AGCTCTCCAAGGTTGTGGCAGG (reverse) for the wild type (500 bp) and GAT CGGCCATTGAACAAGAT (forward) and ATACTTTCTCGGCAGGAGCA (reverse) for the knockout (345 bp). The offspring included wild-type (IDUA+/+) and heterozygous (IDUA+/−) mice.

### Urinary and tissue GAGs assays

Urine was collected and tissue extracts were prepared by sonication in phosphate buffer saline from the wild type (*n* = 4) and IDUA KO (*n* = 4). The tissue extracts were prepared by sonication in phosphate buffer saline. Extracts were centrifuged at 20,000 g for 30 min, and supernatants were collected. GAGs concentrations in urine and tissue were quantified via colorimetric assay (Kamiya Biomedical Co., USA) according to the manufacturer’s instructions, and absorbance was measured at 620 nm using a chondroitin 6-sulfate standard curve. Tissue GAG contents were normalized to the total protein concentration measured using a BCA assay (Pierce, Rockford, IL, USA), and urinary GAG excretion was normalized to creatinine by creatinine assay (Sigma-Aldrich, St Louis, MO, USA).

### Histological analysis of liver and kidney

After the perfusion of the mice with ice-cold normal saline, their tissues were collected and fixed with 4 % paraformaldehyde overnight at 4 °C. The next day, the tissues were embedded in paraffin (Sigma-Aldrich) after dehydration through a 70–100 % ethanol gradient. Finally, the paraffin blocks were cut to a thickness of 4 μm. Hematoxylin-eosin staining of sections was performed according to standard protocols. Images of each section were captured with a digital camera magnifier using a Nikon ECLIPS 80i FL Upright Microscope (Nikon Melville, NY) and were saved as JPEG files.

### X-ray test

Skeletal abnormalities of the mice were examined with an X-ray test using the MX-20 cabinet system (Faxitron, Tucson, AZ, USA) as follows: The mice were anesthetized with intraperitoneal-injected 12.5 mg/kg of Zoletil along with 2.5 mg/kg of Xylazine and further sedated in a 5 % isoflurane chamber for 20 s. The sedated mice were placed on an X-ray tray and posed for lateral, dorsal–ventral, forelimb, and hindlimb views. X-ray films were taken in each at 26 kV for 16 s. The lateral view was imaged at 1x and 3x magnitude for whole body generals and skull details, respectively; the dorsal–ventral view was imaged at 1x and 4x magnitude for whole body generals and skull details, respectively; and the forelimb and hindlimb views were imaged at 5x and 3x magnitude, respectively. In the forelimb and hindlimb images, the tibia length was measured and the foot bones were analyzed for digit numbers and dysmorphology.

### Dual-energy X-ray Absorptiometry test (DEXA)

DEXA testing was conducted to determine the fat contents and bone mineral density of the mouse subjects as follows: The mice (WT; *n* = 5, KO; *n* = 6) were anesthetized as in the X-ray test, positioned for the dorsal–ventral view, including the tail on a DEXA tray, and irradiated four times by X-ray with lower energy (40 kV) and four times with high energy (70 kV) and averaged. Bone mineral density (BMD) and contents (BMC) were analyzed in the whole-skeleton, and femur area and fat content were calculated in the whole-body excluding the head area. The DEXA equipment (Lunar PIXImus) was calibrated with a plastic phantom mouse provided by the manufacturer at the 1320 threshold.

### Open-field test

The explorative activity of the mice was assessed by open-field tests as follows. The mice (WT; *n* = 5, KO; *n* = 6) were allowed to explore an open-top arena (44.5 cm x 44.5 cm) sided with opaque walls (15.0-cm height) and video-recorded for 20 min. The recorded videos were analyzed for exploration distance, moving speed, resting time, the retention time of each subject mouse in the central (zone 3) and peripheral zones (zone 1 and 2), and the number of entries in each zone. The central zone was defined as the enclosed area (31.0 cm x 31.0 cm) at the center of the arena, and the peripheral zone was the rest of the arena, excluding the central zone.

### Rotarod test

The motor balance and learning of the mice was assessed by a rotarod test as follows. The mice (WT; *n* = 5, KO; *n* = 6) were habituated on a rod rotating at 4 rpm for 1 min and subsequently tested at 4–40 rpm for 5 min and recorded for latency at 4–40 rpm. The test was repeated three times with 10-min intervals for recovery for one set of experiments, and three sets of experiments were conducted for three consecutive days with 24-h intervals between each set.

### Grip strength test

The grip strength of mouse paws was measured using a grip strength meter (Bioseb, Vitrolles, France) as follows: The mice (WT; *n* = 5, KO; *n* = 6) were allowed to grip the metal grid of the grip strength meter with their front paws or all four paws while their tails were suspended perpendicular to the grid and subsequently dragged by pulling their tails parallel to the grid until their front paws reached the end of the grid. The test was repeated three times without intervals between each test, and the grip strength data were recorded in g-force units.

### Statistical analysis

All results are expressed as the mean ± SEM. Statistical significance was determined for the compared measurements via a two-tailed *t*-test, followed by Sigmaplot 12.5 (Systat Software, Inc.); p-values of < 0.05 were considered significant.

## Results

### Targeting of IDUA gene and generation of IDUA KO mice

For the targeting of the IDUA gene and generation of IDUA KO mice, ES cells were electroporated with linearized pOSDupDel.Neo-IDUA KO vector, and then plated on fibroblast feeder layers. The ES cells were treated with geneticin (neomycin analog) to select cells that had incorporated the targeting construct. ES cell clones that survived drug selection were screened for homologous recombination events using Southern blot analysis. In the process of constructing the pOSDupDel.Neo-IDUA KO vector, an exogenous *Xho I* restriction site and *Sal I* restriction site were introduced at the 5’ end of the Neo cassette and the 3’ end of the Neo cassette, respectively (Fig. 1a). Therefore, ES cell genomic DNA was digested with *EcoRI* and DNA blots hybridized with a probe corresponding to an IDUA gene region located 5’ and 3’ to the integration site of the construct. With this strategy, the native allele and a mutant allele produced by homologous recombination were indicated. A single clone of the ES cells that had undergone homologous recombination was microinjected into blastocysts, and 10 chimeric mice were generated. Six of the chimeras demonstrated 90 % chimerism by color. Five chimeric males transmitted the mutated allele through the germline. Heterozygote offspring were identified by both PCR and the Southern analysis of the genomic DNA (Fig. 1b, c). Heterozygotes exhibited a grossly normal phenotype and normal fertility. Genotyping 124 offspring from the heterozygote crosses revealed the expected Mendelian ratios (+/+ 37/124, 29.8 %; +/− 54/124, 43.5 % and −/− 33/124, 26.6 %), indicating no significant effect on embryo development. The IDUA KO mice showed coarse facial features and sporadic alopecia, broadened paws, and thickening of the digits and palmar regions in the phenotypic analysis (Fig. 1d).

### Urinary GAGs excretion, tissue GAGs accumulation, and histological analysis

**Fig. 2:**
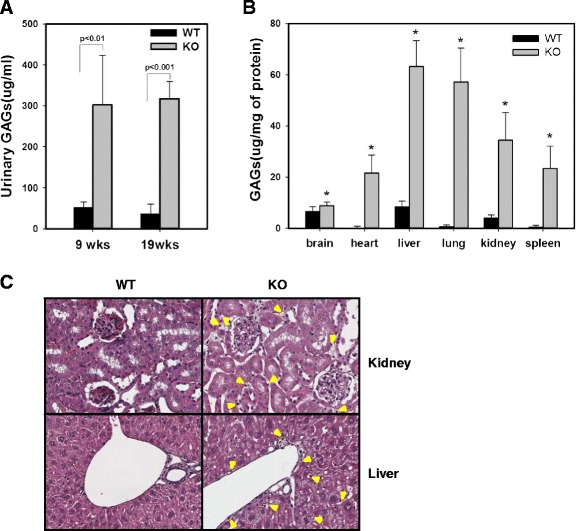
Glycosaminoglycan (GAG) excretion in the urine and accumulation of GAGs in several organs in IDUA KO mice. **a** The urinary excretion of GAGs of 9-week-old (*n* = 4) and 19-week-old (*n* = 4) KO mice were significantly higher than those of wild-type mice at the same ages (*n* = 5). The data were presented as mean ± SEM. **b** GAGs accumulated in the tissues of IDUA KO mice were higher than those of wild-type mice. Asterisk (*) indicates a statistically significant difference (*p* < 0.05). **c** Histological analysis of kidney and liver tissues: By 10 weeks of age, GAGs storage within the reticulo-endothelial system had occurred and hepatocyte vacuoles were noted. Arrow shows vacuoles

Since IDUA plays a role in the degradation of GAGs, such as heparan sulfate and dermatan sulfate, the primary biomarker in the knockout mice is GAGs accumulation. To determine the elevation of GAGs excretion, samples were collected from the wild type and knockout at 9 weeks and 19 weeks for urine and at 22 weeks for tissue. The urine GAGs level was normalized to urine creatinine. The IDUA KO mice showed significantly increased urine GAGs levels at 9 weeks of age (*p* < 0.01) and 19 weeks of age (*p* < 0.001) (Fig. 2a). In addition, the GAGs levels were measured in the brain, heart, liver, lung, kidney, and spleen of wild-type and knockout mice and normalized to total protein (Fig. 2b). In all tissues, GAGs accumulation showed increased level ranges from 1.8- to 90-fold. The histological analysis also showed that many GAGs had accumulated in the lysosomes of the liver and kidney in IDUA KO mice (Fig. 2c). Furthermore, growth and developmental retardation of these organs was detected in IDUA KO mice. In the case of the liver, the following pathological characteristics were shown. Lysosome-laden Kupffer cells were readily found at 4 weeks of age with very little evidence of significant hepatocyte storage. By 10 weeks of age, further progression of GAG accumulation within the reticulo-endothelial system had occurred, and there was evidence of significant hepatocyte vacuolation. At this age, 20 to 30 % of the cytoplasm of the hepatocytes appeared to be taken up by lysosomes, compared with very few discernible lysosomes within normal liver samples, and vacuolization of the cells occurred in the kidney also at 10 weeks of age.

### DEXA assessment and radiographs

Sixteen-week-old male and female IDUA KO mice were collected and scanned for bone density by dual-energy X-ray absorptiometry (DEXA). Femur and whole-bone mineral density (BMD), and bone mineral content (BMC) were assessed (Table 1). Male and female IDUA KO mice had significantly greater femur BMDs compared with age-matched wild-type mice (0.074 ± 0.002 vs. 0.064 ± 0.001, *p* < 0.05 and 0.068 ± 0.002 vs. 0.059 ± 0.002, *p* < 0.01, respectively). In the whole-body analysis, BMD, BMC, and bone area also increased overall in the IDUA KO mice compared with the wild-type mice. However, the tibia length in IDUA KO mice tended to be shorter than that of the age-matched wild type, but it did not reach statistical significance.

**Table 1:** Summary of parameters and statistics for bone mineral analysis by DEXA and tibia length by X-ray in the IDUA KO mice at the age of 16 weeks (mean ± SEM)

	Parameters	Male		Female	
		WT (*n* = 4)	KO (*n* = 4)	WT (*n* = 5)	KO (*n* = 6)
Femur	BMD (g/cm^2^)	0.064 ± 0.001	0.074 ± 0.002****	0.059 ± 0.002	0.068 ± 0.002***
	BMC (g)	0.031 ± 0.001	0.037 ± 0.0002*	0.03 ± 0.0008	0.038 ± 0.001****
	Bone area (cm^2^)	0.48 ± 0.007	0.505 ± 0.006****	0.502 ± 0.018	0.553 ± 0.006
Body	BMD (g/cm^2^)	0.047 ± 0.001	0.053 ± 0.0005**	0.046 ± 0.0004	0.051 ± 0.0006*
	BMC (g)	0.547 ± 0.032	0.755 ± 0.027**	0.0541 ± 0.014	0.700 ± 0.017*
	Bone area (cm^2^)	11.703 ± 0.388	14.233 ± 0.503***	11.806 ± 0.348	13.787 ± 0.237*
	Tibia length (mm)	18.043 ± 0.239	17.555 ± 0.147	18.396 ± 0.110	18.099 ± 0.105

*p < 0.001, **p < 0.005, ***p < 0.01, ****p < 0.05

BMD: Bone mineral density; BMC: Bone mineral content

### Behavioral analysis

**Fig. 3:**
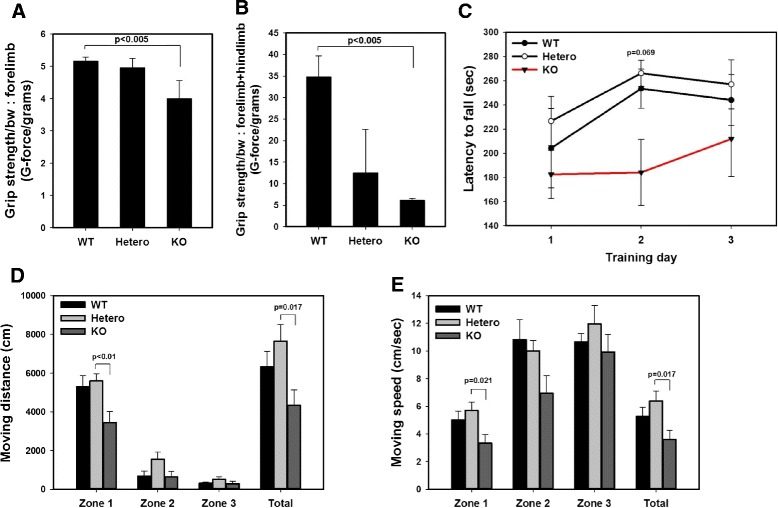
Behavior analysis including grip strength, rotarod, and open-field tests in 9-week-old female IDUA KO mice (*n* = 5). **a**, **b** IDUA KO mice had reduced forelimb strength and reduced forelimb combined with hindlimb grip strength. The data were analyzed by normalizing the mean to body weight. Each mouse was tested three times. **c** Rotarod performance was analyzed daily for 3 days. Although no significant difference was observed, there was a decrease of latency to fall tendency in IDUA KO mice. **d**, **e** In the open-field test, IDUA KO mice traveled shorter distances and more slowly compared with wild-type mice. Zones 1 and 2 are located at peripheral area, and zone 3 represents central zone

We quantified the grip strength, latency on rotarod, and open-field activity of 8- or 9-week-old IDUA KO mice. As shown in Fig. 3a and b, both the forelimbs and hindlimbs were significantly impaired in the IDUA KO mice (*p* < 0.005, respectively). Defects in motor function were confirmed on the rotarod and in the open field, in which there was a significant decrease in rearing activity. During the training day, the IDUA KO mice showed the shortest latencies to fall compared with their control littermates (Fig. 3c). The IDUA KO mice spent less time in the peripheral zones compared with the wild-type mice (Fig. 3d, e).

In the open-field test, the KO mice showed less exploratory behavior. They spent significantly less time exploring (Fig. 3d), and the moving speed was significantly decreased in the KO mice, especially in the peripheral zone (Fig. 3e). Thus, all of these results suggest that the IDUA KO mouse has defects in motor function, which affect both the quality and quantity of activity.

### Statistical analysis

The statistical analysis for all experiments was performed using the two-sample t-test. P-values of < 0.05 were considered significant. If equal variance failed, the Mann–Whitney rank sum test was used. The error bars refer to mean ± SEM.

## Discussion

In this study, we observed a decrease in grip function, decreased performance in the rotarod test, and hypo-activity in the open-field test in the IDUA knockout mouse, which mimic the limitations of joint mobility and decreased motor performance in 6-min walk tests in patients with MPS I. Furthermore, these behavioral tests and parameters can be used in evaluating the efficacy of the therapeutic modalities in MPS I. Decrease in grip function in mice is consistent with the severe limitation of the joint function caused by claw-hand deformity and/or carpal tunnel syndrome in patients with MPS I. Decreased performance in the rotarod test, which reflects motor balance and learning, and hypo-activity in the open-field test are also consistent with decreased performance in 6-min walk tests, which is the gold standard test for the evaluation of the efficacy of therapy in patients with MPS I. Dr. Muenzer reported that the skeletal changes of MPS I patients result in profound loss of joint range of motion and restricted mobility. The hands and wrists of patients show decreased range of wrist motion, stiffening of the IP joint, and curved finger [19].

Animal models are required for therapeutic drug development; therefore, the MPS I-type murine model has been generated and reported in addition to naturally occurring MPS I disease-type animals [8–16] (Table 2). Here, we report the generation of an IDUA knockout mouse produced by gene targeting the disruption of the murine IDUA gene using homologous recombination; our IDUA KO mice have a large deletion from exons 6 and 9. This mouse model showed increased urinary GAGs excretion and tissue GAGs accumulation (Fig. 2), similar to the previously described results (Table 2). It has been known that GAGs accumulate in all the organs because Hurler syndrome is a systemic disorder caused by genetic mutation [20]. There are several reports showing the accumulation of GAGs in organs [21–24]. It has been well known that the liver and kidney are organs representative of the accumulation of quite a large amount of GAGs [20]. Moreover, these organs showed the dramatic effect of the diminution of GAGs after enzyme replacement therapy in patients with Hurler syndrome and an animal study [17, 18, 25, 26].

**Table 2:** Hurler syndrome mouse model in the literature and the present study

Deleted locus	Properties	Reference
Exon 6	• Targeted disruption	[14], Clarke K
	• No detectable IDUA enzyme activity	
	• Increased urinary GAGs	
	• Flattened facial profile	
	• Thickening of the digits	
Exon 6	• Targeted disruption	[13], Ohmi K
	• No phenotype data	
Between exon 8 and 9	• Knock-in mouse that carries IDUA-W402X mutation	[15], Wang D
	• MPS I-H	
	• No detectable IDUA enzyme activity	
	• Increased GAGs in the urine and tissues	
From a part of exon 6 to a part of exon 9	• Targeted disruption	This study
	• Increased urine GAGs excretion	
	• Increased BMD and BMC	

We observed significant differences in the bone mineral density and bone mineral content of the femur and whole bone in DEXA analysis. The IDUA KO mice showed increased BMD in both the males and females compared with the wild-type mice (Table 1).

It is well known that MPS I patients have skeletal abnormalities (termed dysostosis multiplex), including enlarged skull, short and thick thorax, brachydactyly, and irregular carpal bones in the hands [5, 19, 27–29]. The clavicles are short, thickened, and irregular. The long bones are short with wide shafts. The endochondral growth plates are thickened and distorted. Typically, the pelvis is poorly formed. The femoral heads are small, and the coxa valga is common. Severe joint deformity and stiffness are common [30]. Although dysostosis multiplex was hard to determine in the KO mouse because of the tiny size of its individual bones, IDUA KO mice tend to have broad and short bone in radiograph (Additional file 1: Figure S1). These findings coincide with reported MPS I mouse model [14–16].

Some previous studies showed that BMD increased in the mouse model of MPS I, like the clinical features in human MPS I patients [31, 32]. Wang reported that BMD was increased in 35-week-old mice [15]. Garcia-Rivera et al. also described eight-month-old NOD-SCID-MPS-I (IDUA^null^: MPS-I) mice with thickening of the zygomatic arch of the skull [13]. In the present study, the IDUA KO mice showed these findings at 16 weeks, which is consistent with previous reports [15, 24, 25], but the change was noted at a younger age.

MPS I-H patients show developmental delay and a decrease in intellectual capacity. Neuropsychological manifestations may arise from primary GAGs accumulation in the central nervous system or be caused by deposits in adjacent structures, such as the meninges and bone structures [33]. Besides central nervous system dysfunction, peripheral nerve abnormalities and muscle contracture may account for some of the manifestations, such as carpal tunnel syndrome, observed in patients with MPS I-H. Initial subtle clinical signs of carpal tunnel syndrome in MPS I patients include alterations in grasp patterns, increasing difficulty with fine motor tasks, and withdrawal of the hands from the touch of others [34]. In this study, we admit that the joint and locomotion disturbance in the KO mouse may have partly resulted from the dysfunction of the central and peripheral nervous system.

Lastly, we want to mention that the open-field test measures the activity level of the animal [35]. The open-field box consisted of a black square box, in which each animal was placed for ten minutes. The overall distance traveled by the IDUA KO mice was significantly shorter than that traveled by the wild-type or heterozygous mice, which also showed a slower moving speed. The IDUA KO mice had a shorter traveling distance and decreased moving distance (Fig. 3d, e), suggesting that IDUA KO mice are hypoactive, and this hypo-activity can be quantitated in open-field testing. The severe developmental effects in children with MPS I are also associated with placid rather than aggressive behavior [33]. Animal models are helpful to understand the disease biology and pathophysiology, but the behavioral aspects are much more complex and species-specific. Despite these limitations, combining these tests in a murine model will be a valuable tool for the development of therapeutics and the study of pathologic behavior for patients with MPS type I.

## Conclusions

We generated a new IDUA KO mouse, tested open field, rotarod, and grip strength and demonstrated a decrease in grip strength, decreased performance, and hypo-activity, which may be useful for investigating therapeutic approaches and studying the pathogenesis of joint and locomotion symptoms in MPS I.

## Additional file

Additional file 1: Figure S1. Radiograph of IDUA KO and wild type mice. Arrows show the area where thickness of the bone is evident. (PPTX 440 kb)

## Abbreviations

MPS I: Mucopolysaccharidosis type I
MPS I-H: Hurler syndrome
MPS I-S: Scheie syndorme
MPS I-HS: Hurler-Scheie syndrome
IDUA: Alpha-L-iduronidase
KO: Knockout
DEXA: Dual-Energy X-ray Absorptiometry analysis
BMD: Bone Mineral Density
BMC: Bone Mineral Contents
ERT: Enzyme Replacement Therapy
GAGs: Glycosaminoglycans
ES: Embryonic Stem
