# Supplementary figures and images for: Decreased performance in IDUA knockout mouse mimic limitations of joint function and locomotion in patients with Hurler syndrome
Source: Orphanet J Rare Dis. 2015 Sep 25;10:121. doi: 10.1186/s13023-015-0337-3 (PMC4582722; doi:10.1186/s13023-015-0337-3)

## Slide 1
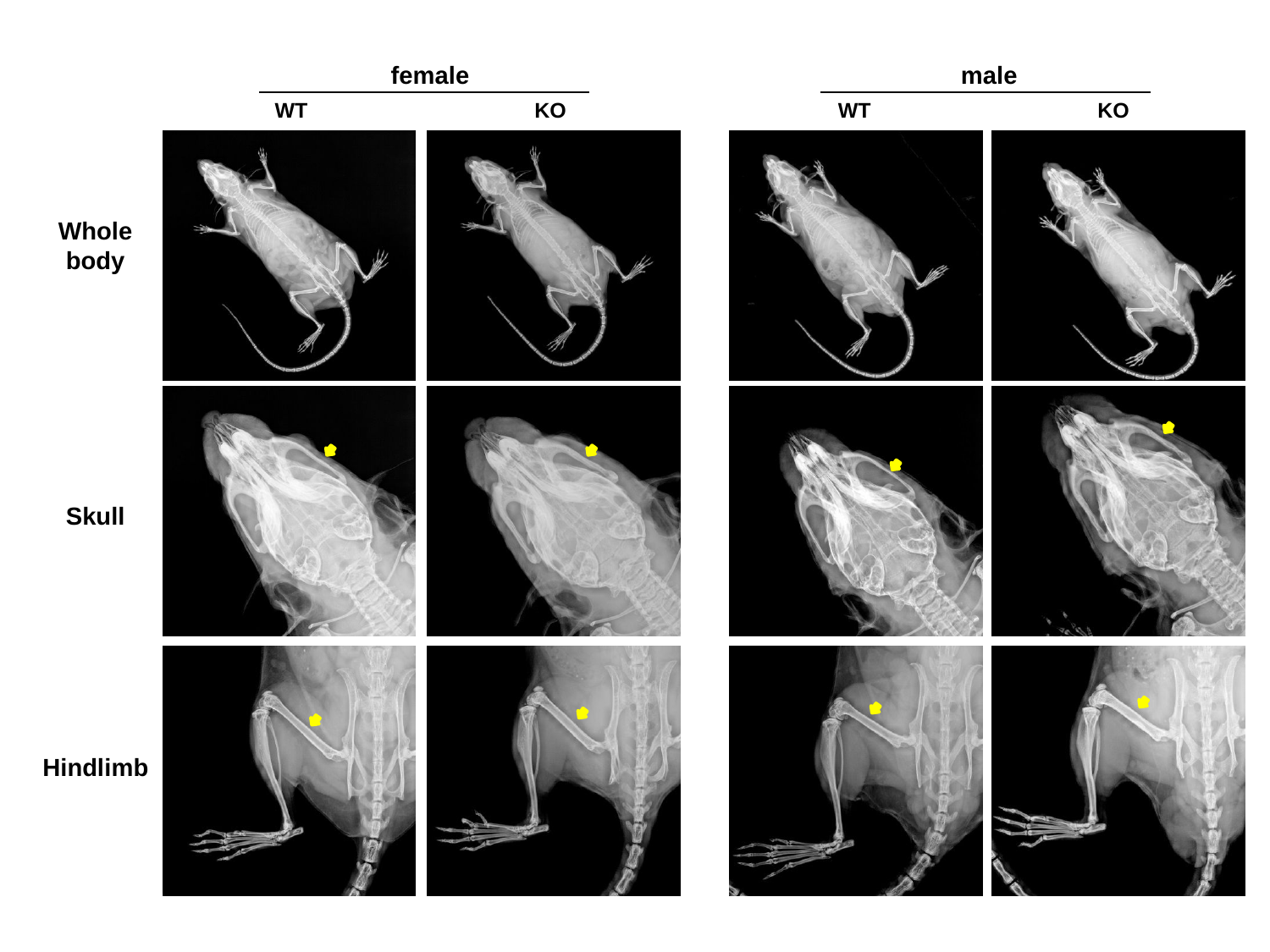

female
male
WT
KO
WT
KO
Whole
body
Skull
Hindlimb

Supplement: Additional file 1: Figure S1. — Radiograph of IDUA KO and wild type mice. Arrows show the area where thickness of the bone is evident. (PPTX 440 kb) [file 13023_2015_337_MOESM1_ESM.pptx]
